# Single-Nanoparticle Electrochemical Collision for Monitoring Self-Assembly of Thiol Molecules on Au Nanoparticles

**DOI:** 10.3390/bios14080393

**Published:** 2024-08-15

**Authors:** Yiyan Bai

**Affiliations:** Department of Chemistry, Yuncheng University, Yuncheng 044000, China; byy@ycu.edu.cn; Tel.: +86-35-9251-3098

**Keywords:** nanoparticles, self-assembly, single-particle detection

## Abstract

A precise understanding of the self-assembly kinetics of small molecules on nanoparticles (NPs) can give greater control over the size and architecture of the functionalized NPs. Herein, a single-nanoparticle electrochemical collision (SNEC)-based method was developed to monitor the self-assembly processes of 6-mercapto-1-hexanol (6-MCH) and 1-hexanethiol (MCH) on Au NPs at the single-particle level, and to investigate the self-assembly kinetics exactly. Results showed that the self-assembly processes of both consisted of rapid adsorption and slow recombination. However, the adsorption rate of MCH was significantly lower than that of 6-MCH due to the poorer polarity. Also noteworthy is that the rapid adsorption of 6-MCH on Au NPs conformed to the Langmuir model of diffusion control. Hence, the proposed SNEC-based method could serve as a complementary method to research the self-assembly mechanism of functionalized NPs.

## 1. Introduction

The use of small molecules such as amino acids and thiol compounds to form self-assembled monolayers (SAMs) on the surface of nanoparticles (NPs) is a common method to obtain functionalized NPs for sensing, catalysis, molecular recognition and molecular electronics [1,2,3,4,5]. Therefore, it is necessary to accurately monitor the self-assembly processes of small molecules on the surface of NPs by detecting assemblies in solution at the single-particle level. However, sample pretreatments and vacuum operation processes of traditional electron microscopy methods can greatly affect the true morphology of the assemblies. At this time, some in situ characterization techniques have been developed, such as liquid-phase transmission electron microscopy (LP-TEM, JEOL, Tokyo, Japan) [6,7], small angle X-ray scattering (SAXS, Forvis Technologies, Santa Barbara, CA, USA) [8,9], and dynamic light scattering (DLS, Malvern, UK) [10]. However, the purchase and maintenance costs of LP-TEM are relatively high, making it difficult to promote their applications. SAXS and DLS show the average scattered light intensity of multiple particles. As the scattering power is inversely proportional to the sixth power of the radius for spherical NPs [11,12], it is not suitable for characterizing size polydisperse samples generated in the self-assembly processes.

With the development of micro/nano processing technology and analytical methods, it is possible to detect dispersed NPs in solution at the single-particle level. Among them, single-nanoparticle electrochemical collision (SNEC) has attracted researchers’ attention due to their advantages of high sensitivity, high throughput, fast response, and low cost [13,14,15,16,17,18,19,20]. Based on different reaction modes, SNEC can be classified as electrocatalytic amplification (ECA) [21], particle self-electrolysis [22], diffusion blocking [23], and capacitive impact [24]. The principle of ECA is based on inner-sphere heterogeneous electrode reactions (ISERs). Specifically, in ISERs (e.g., hydrogen evolution reaction), the reactants, products, and intermediates interact strongly with the electrode surface, so the reaction performance is closely related to the electrode surface structure. Due to the significant difference in surface structure between the substrate electrode and the NPs, although there is almost no Faraday reaction of the electroactive substance on the substrate electrode at a specific voltage, when a single NP collides with the substrate electrode, it can catalyze the reaction of the electroactive substance as a nanoelectrode, resulting in a current transient corresponding to the single NP [25,26]. Recently, SNEC has been used to monitor the self-assembly process of nanomaterials. For example, Zhang et al. utilized SNEC to, in real-time, monitor the aggregation of α-Synuclein (α-Syn), and found that vitamin D (VD) can reduce the probability of early aggregation of α-Syn [27]. Compton et al. investigated the effect of magnetic fields on the aggregation of Fe_3_O_4_ NPs based on SNEC, and showed that even in high salt environments, Fe_3_O_4_ NPs did not undergo significant aggregation when there was no magnetic field [28].

**Scheme 1:**
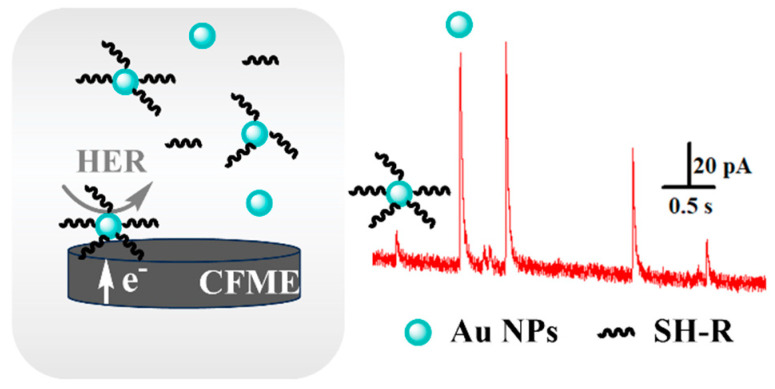
SNEC-based method for monitoring the self-assembly processes of thiol molecules on the surface of Au NPs.

Herein, Au–S bonding was used to modify thiol small molecules such as 6-mercapto-1-hexanol (6-MCH) and 1-hexanethiol (MCH), on the surface of Au NPs. Based on the influence of the electroactive area of Au NPs on the current intensity, the dynamic processes of the self-assembly of thiol molecules on Au NPs were monitored in solution at the single-particle level (Scheme 1).

## 2. Materials and Methods

### 2.1. Self-Assembly of Thiol Molecules on Au NPs

Au NPs were synthesized following a modifying sodium citrate reduction method [29], and the average diameter was found to be 40 nm (Figure S1). The stocked Au NP solution was washed with ultrapure water three times by ultrafiltration (4000 rpm, 2 min), and the concentration was about 60 pM. To achieve the self-assembly of thiol molecules on Au NPs, after adding 10 µL of a certain concentration of thiol molecules to 100 µL of Au NPs (60 pM), the mixture was eddied at room temperature for a period of time.

### 2.2. SNEC Tests for Self-Assembled Au NPs

After adding 110 µL of the above self-assembled Au NPs to 10 mL of 0.8 mM HClO_4_, the mixture was sonicated for 30 s to disperse uniformly for the further SNEC tests which were conducted according to our previous work [30]. Specifically, a conventional three-electrode setup with a carbon fiber microelectrode (CFME, *d* = 7 µm, Figure S2) as a working electrode [31], Ag/AgCl (3 M KCl) as reference electrode, and a Pt wire as a counter electrode were used. The spikes were recorded and magnified by an EPC-10 patch-clamp amplifier (HEKA Electronics, Stuttgart, Germany), and the signals were sampled at 10 kHz. The amplifier’s internal low-pass Bessel filter and I_Bessel filter was set to 10 kHz and 0.1 KHz, respectively. The potential was held at −0.6 V where the CFME is inert toward hydrogen evolution reaction and the current intensity and collision frequency of Au NPs are the highest. Additionally, all collision experiments were performed in a Faraday cage within 30 min to minimize agglomerate effects [17].

## 3. Results and Discussion

### 3.1. Feasibility of Self-Assembly Detection of Thiol Molecules on Au NPs Based on SNEC

**Figure 1:**
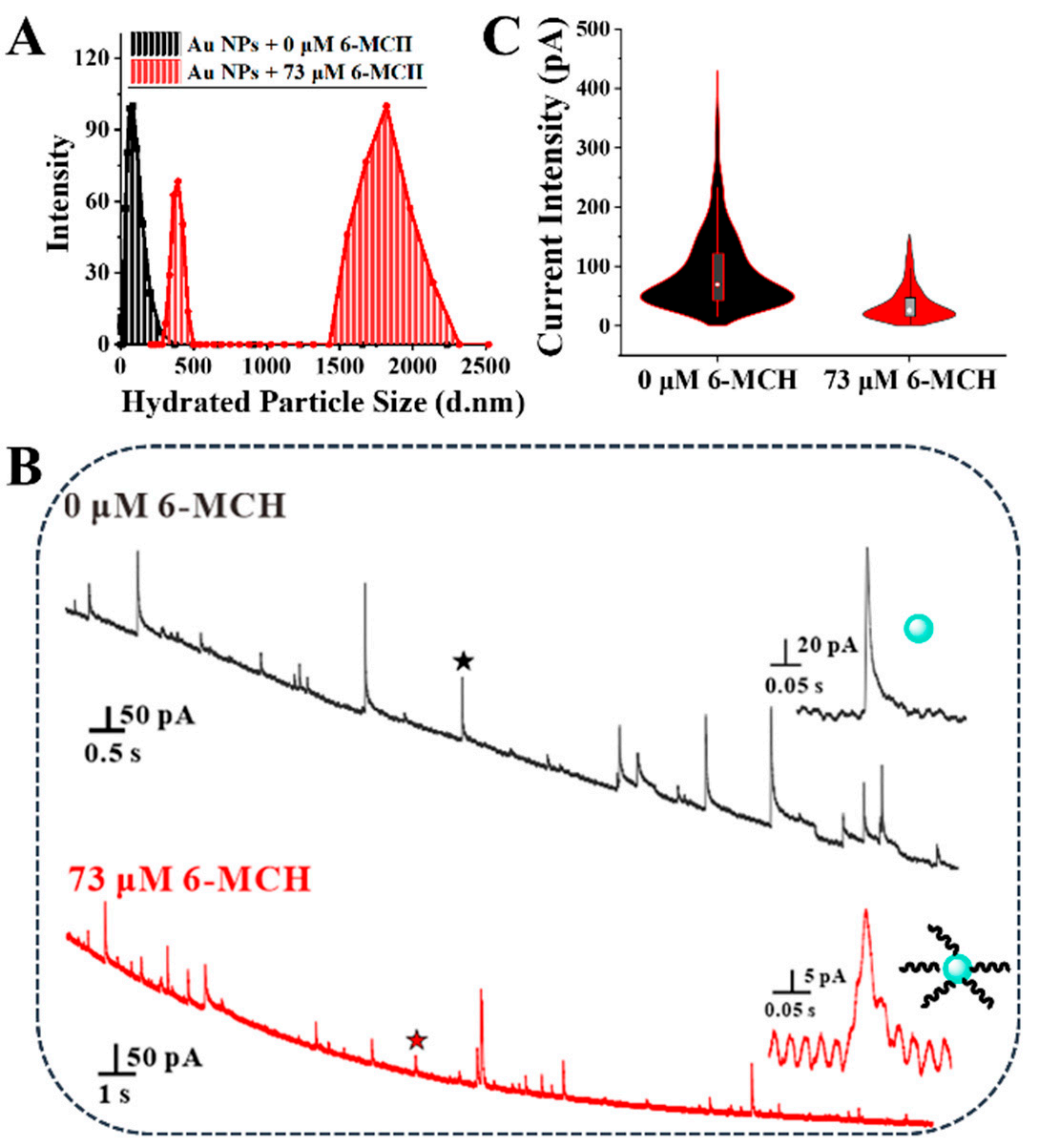
Comparisons of hydrated particle size (**A**) chronoamperometry curves (**B**) and current intensity (**C**) before and after 6-MCH added into Au NPs. The inserts in (**B**) are typical current transients measured before (black star) and after (red star) 6-MCH added into Au NPs.

To investigate the feasibility of self-assembly, DLS characterizations were performed before and after injection of 6-MCH into Au NPs solutions. It was found that the hydrated particle size and polydispersity (PDI) were significantly increased after injection of 6-MCH (Figure 1A), indicating that 6-MCH successfully self-assembled on Au NPs. Considering that the monodispersing of NPs in collision experiments is vital for the self-assembly detection of thiol molecules on Au NPs based on SNEC, the stability of Au NPs in 0.8 mM HClO_4_ solution was investigated. The current intensity, which was measured before and after Au NPs dispersed in 0.8 mM HClO_4_ for 30 min, had essentially the same distributions, indicating that Au NPs can basically exist stably in 0.8 mM HClO_4_ for 30 min (Figure S3). Hence, SNEC tests were conducted for the above two samples within 30 min to avoid agglomerative effects, and peak-type current transients with an average current intensity of 95 pA appeared when only Au NPs existed in the system (Figure 1B,C). Whereas, after Au NPs and 6-MCH (73 μM) co-incubation at room temperature for 10 min, the average current intensity markedly decreased to 37 pA. Considering that the cyclic voltammetry (CV) of CFME in 0.8 mM HClO_4_ before and after 6-MCH added into Au NPs solutions were similar (Figure S4), the interaction between CFME and 6-MCH was ignored. So, the relatively noticeable distinction in current intensity caused by 6-MCH occupied some active sites of Au NPs [29], indicating that SNEC can detect the self-assembly of thiol molecules on Au NPs sensitively.

### 3.2. Effect of the C_6-MCH_/C_Au NPs_ on the Assembly Efficiency

**Figure 2:**
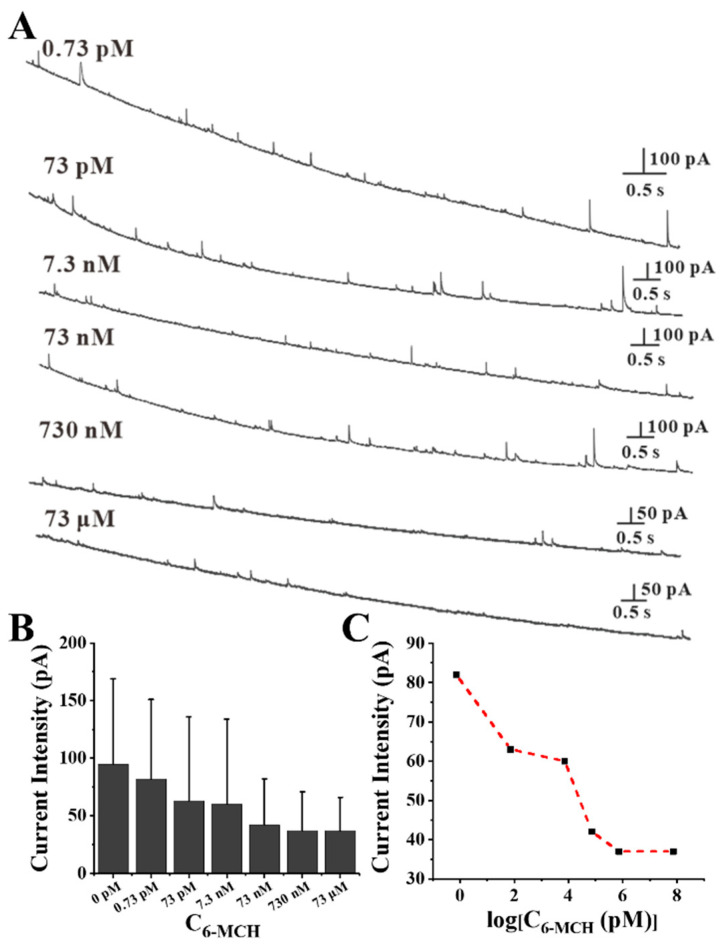
(**A**) Chronoamperometry curves of Au NPs’ assemblies with different concentrations of 6-MCH. (**B**) Current intensity of Au NPs assemblies with different concentrations of 6-MCH. (**C**) The curve of current intensity changing with log[*C*_6-MCH_].

The effect of the *C*_6-MCH_/*C*_Au NPs_ on the assembly degree was explored with 60 pM of Au NPs and a relatively wide range of 6-MCH concentrations, from 0.73 pM (*C*_6-MCH_/*C*_Au NPs_ = 1.2 × 10^−2^) to 73 μM (*C*_6-MCH_/*C*_Au NPs_ = 1.2 × 10^6^). After co-incubation at room temperature for 10 min, more small signals appeared with the increase of the concentration of 6-MCH (Figure 2A), coinciding with the current intensity decrease with the increasing of the concentration of 6-MCH (Figure 2B). As shown in Figure 2C, the average current intensity decreased to the lowest value (*i*_p_ ≈ 37 pA) when *C*_6-MCH_ = 730 nM (*C*_6-MCH_/*C*_Au NPs_ = 1.2 × 10^4^), suggesting that the self-assembly degree of Au NPs reached its highest at this point.

### 3.3. Self-Assembly Monitoring of Thiol Molecules on Au NPs Based on SNEC

**Figure 3:**
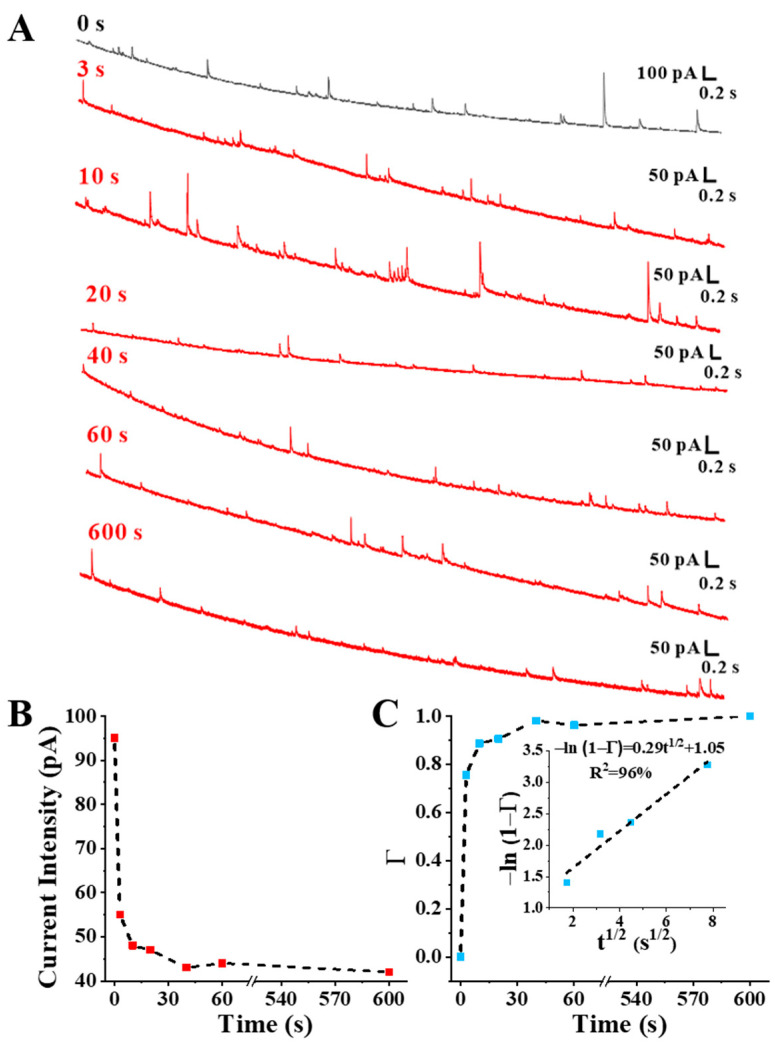
Chronoamperometry curves (**A**), current intensity (**B**), and surface coverage (**C**) of Au NP assemblies with 6-MCH (*C*_6-MCH_/*C*_Au NPs_ = 1.2 × 10^3^) at different times. The insert showed the curve of −ln(1 − *Γ*) changing with *t*^1/2^.

As shown in Figure 3A, the self-assembly process of 6-MCH on Au NPs was monitored by SNEC under the condition of *C*_6-MCH_/*C*_Au NPs_ = 1.2 × 10^3^, and the uneven peak-type current transients were caused by the heterogeneity of Au NPs and self-assembly efficiency of 6-MCH on Au NPs. Figure 3B showed that the current intensity significantly decreased when the incubation time was only 3 s, then the current intensity slowly decreased with the extension of incubation time. To further investigate the kinetics of the assembly process, we converted the current intensity into the percentage of adsorbed species in the entire area of Au NPs (*Γ*) by Equation (1).

(1) $$\Gamma =\frac{i_{p,t}{-i}_{p,t=0}}{i_{p,t=600}{-i}_{p,t=0}}$$

where *i_p,t_*_=0_ is the current intensity of the bare Au NPs, and *i_p,t_* is the current intensity measured at one time point in the self-assembly process. The current intensity measured at the last time, *i_p,t_*_=600_, was used as the value when Au NPs was completely covered by 6-MCH.

As shown in Figure 3C, two regions of change in *Γ* were observed during the self-assembly process: the rapid assembly process before 3 s, and the gradual increase in surface coverage to one over a longer period of time. This indicated that the self-assembly of 6-MCH on Au NPs consists of two processes: rapid adsorption and slow recombination [32,33]. Au–S bonds were rapidly formed between 6-MCH and Au NPs in solution through chemical adsorption [34]. At this time, 6-MCH has no orientation on the surface of Au NPs, causing a large amount of 6-MCH to quickly adsorb disorderly on Au NPs. After that, due to the van der Waals force between the 6-MCH molecules [35], the chemisorbed 6-MCH molecules slowly rearranged from an irregular configuration to an upright configuration, forming an ordered and dense SAM. In addition, we also found that t^1/2^ was linearly associated with −ln(1 − *Γ*), −ln(1 − *Γ*) = 0.29*t*^1/2^ + 1.05 (R^2^ = 96%), indicating that the rapid adsorption of 6-MCH on Au NPs coincided with the Langmuir model of diffusion control Equation (2) [36].

*Γ* = 1 − exp(−k_a_*ct*^1/2^) (2)

where k_a_ is the adsorption kinetic constant, and c is the concentration of 6-MCH molecules. k_a_ was calculated to be 3.97 × 10^6^ L/mol, which was of the typical order of magnitude for the alkanethiol systems [37].

### 3.4. Effect of Thiol Molecular Structure on the Packing Efficiency of SAMs

**Figure 4:**
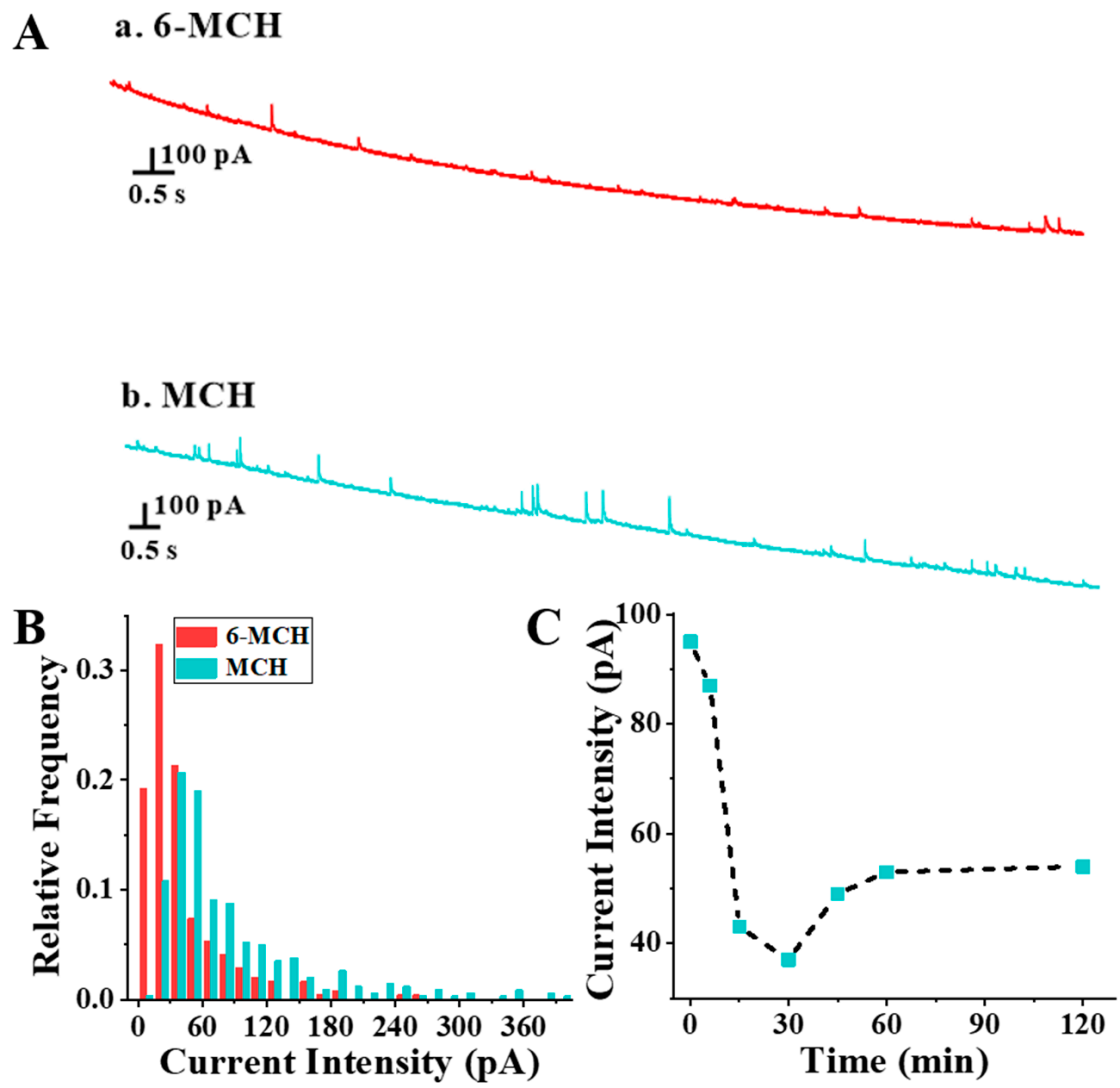
Chronoamperometry curves (**A**) and frequency distributions of current intensity (**B**) of Au NPs assemblies with 6-MCH and MCH. (**C**) Current intensity of Au NPs assemblies with MCH (*C*_MCH_/*C*_Au NPs_ = 1.2 × 10^3^) at different times.

To further illustrate the effect of thiol molecular structure on the packing efficiency of SAMs, the self-assembly results of 6-MCH and MCH on Au NPs were compared under the same conditions (*C*_6-MCH or MCH_/*C*_Au NPs_ = 1.2 × 10^3^, *t* = 6 min). As shown in Figure 4A,B, the current intensity of MCH-functionalized Au NPs was significantly larger than that of 6-MCH-functionalized Au NPs, indicating that the assembly efficiency of MCH on Au NPs was lower than that of 6-MCH. The hydrated particle size of MCH-functionalized Au NPs being smaller than that of 6-MCH-functionalized Au NPs also proved this (Figure S5). Considering that the polarity of the solvent has already been proved to be related to the packing efficiency of SAMs [3], it can be hypothesized that the different polarity of 6-MCH and MCH caused the discernible SAM packing efficiencies. Moreover, a similar trend was observed in the self-assembly process of MCH on Au NPs (*C*_MCH_/*C*_Au NPs_ = 1.2 × 10^3^, Figure 4C and Figure S6): a rapid assembly process before 30 min, and a slow recombination process after 30 min, where the current intensity slowly increased with the prolongation of incubation time. The reason might be the reorganization of disorderly adsorbed MCH, which transited from irregular to upright configurations, resulting in an increase in exposed Au atoms.

## 4. Conclusions

In summary, SNEC-based method was developed to monitor the self-assembly processes of thiol small molecules on Au NPs in solution at the single-particle level. Results showed that both the self-assembly of 6-MCH and MCH on Au NPs contained two processes: rapid adsorption and slow recombination. However, the assembly efficiency of MCH was significantly lower than that of 6-MCH due to the poorer polarity. Further, the Langmuir model of diffusion control was proved to be suitable for the rapid assembly process of 6-MCH Au NPs, and the calculated adsorption kinetic constant (k_a_ = 3.97 × 10^6^ L/mol) was correspondent with the reported value for the alkanethiol systems. Therefore, the proposed SNEC-based method holds great potential to investigate the self-assembly mechanism of small molecule functionalized NPs in solution at the single-particle level, and provide effective guidance for the design and synthetise of functionalized NPs. Considering that the shape of current transients is informative in particle properties, further exploration of current shape-based SNEC sensors should be carried out to effectively study the self-assembly mechanism.

## Data Availability

Data are contained within the article.

## Supplementary Materials

The following supporting information can be downloaded at: https://www.mdpi.com/article/10.3390/bios14080393/s1, Figure S1: TEM images for the synthesized Au NPs, and corresponding histograms showing statistical size distributions determined from TEM images (*n* = 300); Figure S2: SEM images of disk CFME (*d* = 7 µm), and cyclic voltammograms of 5.0 mM K_3_Fe(CN)_6_/0.1 M KCl with a 50 mV/s scan rate for CFME; Figure S3: Current intensity of Au NPs (*d* = 40 nm) after being added into 0.8 mM HClO_4_ solution for different times; Figure S4: Cyclic voltammetry scans of CFME in 0.8 mM HClO_4_ before and after 6-MCH was added into Au NPs solutions; Figure S5: Hydrated particle size of 6-MCH-functionalized Au NPs and MCH-functionalized Au NPs; Figure S6: Violin plots of current intensity of Au NP assemblies with MCH (*C*_MCH_/*C*_Au NPs_ ≈ 1.2 × 10^3^) at different times.
