# Supplementary material for: Single-Nanoparticle Electrochemical Collision for Monitoring Self-Assembly of Thiol Molecules on Au Nanoparticles
Source: Biosensors (Basel). 2024 Aug 15;14(8):393. doi: 10.3390/bios14080393 (PMC11353042; doi:10.3390/bios14080393)
Supplement: Supplementary file 1 [file biosensors-14-00393-s001.zip › biosensors-3107340-supplementary.pdf]

Supporting Information

Single-Nanoparticle Electrochemical Collision for Monitoring Self-Assembly of Thiol Molecules on Au Nanoparticles

Yiyan Bai

Department of Chemistry, Yuncheng University, Yuncheng 044000, China; byy@ycu.edu.cn; Tel.: +86-359-2513098; Fax: +86-359-2513059.

Table of Contents

S1. Reagents and instruments. .... - 2 -

S2. Characterizations of gold nanoparticles (Au NPs). .... - 2 -

S3. Characterizations of carbon fiber microelectrode (CFME). .... - 2 -

S4. Characterizations of the stability of Au NPs in 0.8 mM $HClO_4$ solution. .... - 3 -

S5. Study on the interaction between CFME and 6-MCH. .... - 4 -

S6. Comparison of hydrated particle size of 6-MCH-functionalized Au NPs and MCH-functionalized Au NPs. .... - 4 -

S7. Comparison of current intensity of Au NPs assemblies with MCH ($C_{MCH}/C_{Au\ NPs} \approx 1.2 \times 10^3$) at different time. .... - 5 -

S1. Reagents and instruments.

All chemicals were of analytical grade and used as received. 6-Mercapto-1-hexanol (6-MCH, 97%) and 1-Hexanethiol (MCH, 96%) were purchased from Aladdin and Sigma-Aldrich respectively. Sodium citrate, perchloric acid ($HClO_4$, 70%), chloroauric acid ($HAuCl_4$, 1%) were purchased from Sinopharm Chemical Reagent Co., Ltd. (Shanghai, China). Carbon fiber (*d*=7 μm) was purchased from Goodfellow Co. (Oxford, U.K.). Scanning electron microscope (SEM) measurements were performed on a S-4800 instrument (Hitachi, Japan).

The collision experiments were performed in a Faraday cage within 30 min to minimize agglomerate effects[1], and the chronoamperometry spikes were recorded and magnified by an EPC-10 patch-clamp amplifier (HEKA Electronics, Germany). The potential was held at -0.6 V vs Ag/AgCl (3 M KCl), and the signals were sampled at 10 kHz, bessel and I_bessel filtered at 10 KHz and 0.1 KHz respectively. The cyclic voltammogram (CV) tests were performed on a CHI 660E electrochemical workstation and a CHI 200B Faraday cage (CH Instruments, Inc. Shanghai, China).

S2. Characterizations of gold nanoparticles (Au NPs).

Au NPs were synthesized following a modifying sodium citrate reduction method[2]. Specifically, 0.5 mL of 1% (w/v) $HAuCl_4$ was added to 50 mL ultrapure water and heated to 95 °C under stirring. After 5 min, 0.3 mL 1% sodium citrate was added and continued reaction for 15 min. The stocked Au NPs solution were washed with ultrapure water three times by ultrafiltration (4,000 rpm, 2 min) for later use, and the average diameters were found to be 40 nm (Figure S1).

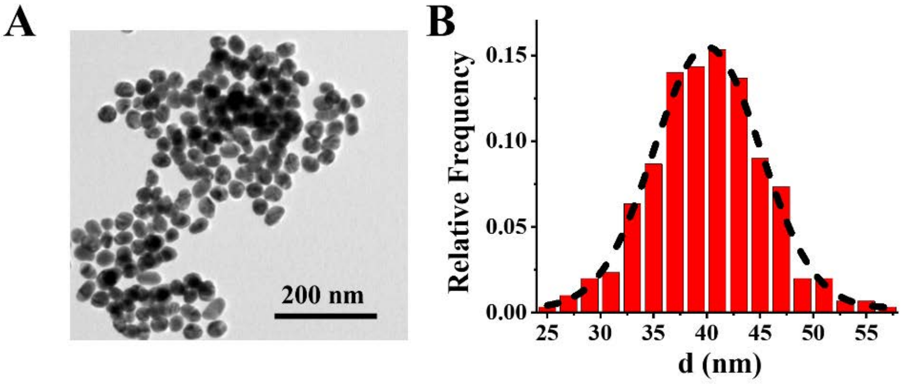

Figure S1. TEM images for the synthesized Au NPs, and corresponding histograms showing statistical size distributions determined from TEM images (*n*=300).

S3. Characterizations of carbon fiber microelectrode (CFME).

As shown in Figure S2A, CFMEs were fabricated by heat-sealing carbon fiber (*d*=7 μm) in borosilicate glass capillaries (1.15 mm OD; 1.0 mm ID)[3]. The prepared CFMEs were soaked in a piranha solution for about 20 s, then rinsed with ultrapure water for later use. With 5.0 mM $K_3Fe(CN)_6$/0.1 M KCl (D=7.6×10^{-6} cm^2/s) afforded limiting current plateaus of approximately 5 nA (Figure S2B)[4], the real aera of CFME was determined to be 34 μm^2.

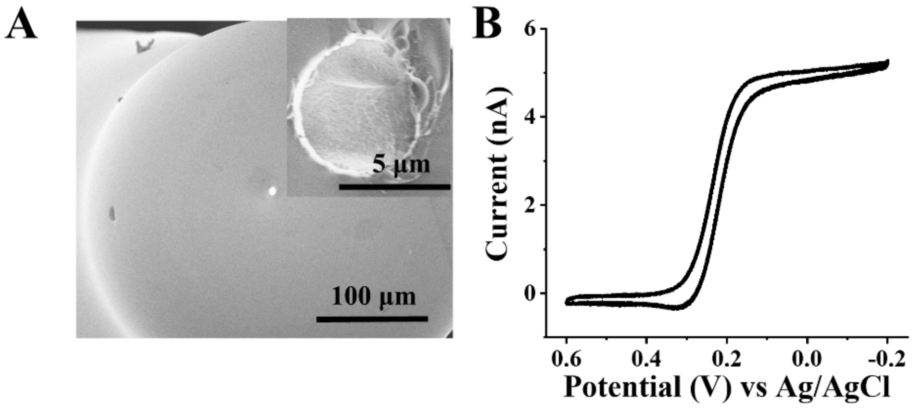

Figure S2. (A) SEM images of disk CFME (*d*=7 µm), the insert was the high-magnification SEM image of CFME. (B) Cyclic voltammograms of 5.0 mM $K_3Fe(CN)_6$/0.1 M KCl with a 50 mV/s scan rate for CFME.

S4. Characterizations of the stability of Au NPs in 0.8 mM $HClO_4$ solution.

Considering that the monodispersion of NPs in collision experiments is vital for the self-assembly detection of thiol molecules on Au NPs based on SNEC, the stability of Au NPs in 0.8 mM $HClO_4$ solution was investigated. As shown in Figure S3, current intensity measured before and after Au NPs dispersing in 0.8 mM $HClO_4$ for 30 min had essentially the same distributions, indicating that Au NPs can basically exist stably in 0.8 mM $HClO_4$ for 30 min. Hence, all collision experiments were performed in 0.8 mM $HClO_4$ within 30 min to avoid agglomerate effects.

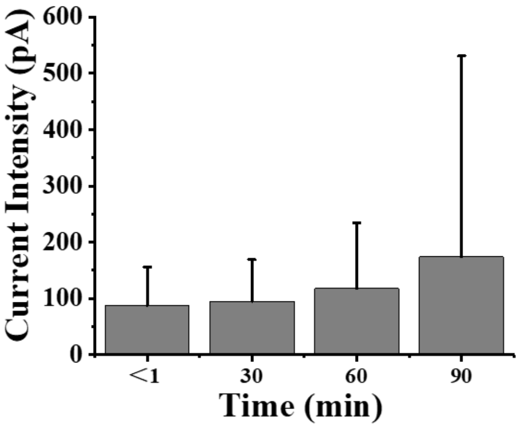

Figure S3. Current intensity of Au NPs (*d*=40 nm) after added in 0.8 mM $HClO_4$ solution for different time.

S5. Study on the interaction between CFME and 6-MCH.

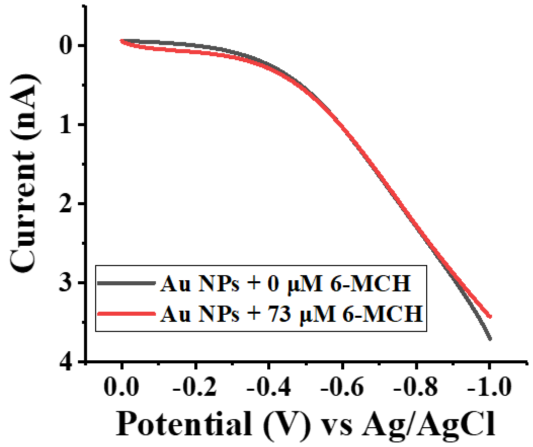

Figure S4. Cyclic voltammetry scans of CFME in 0.8 mM $HClO_4$ before and after 6-MCH added into Au NPs solutions.

S6. Comparisons of hydrated particle size of 6-MCH-functionalized Au NPs and MCH-functionalized Au NPs.

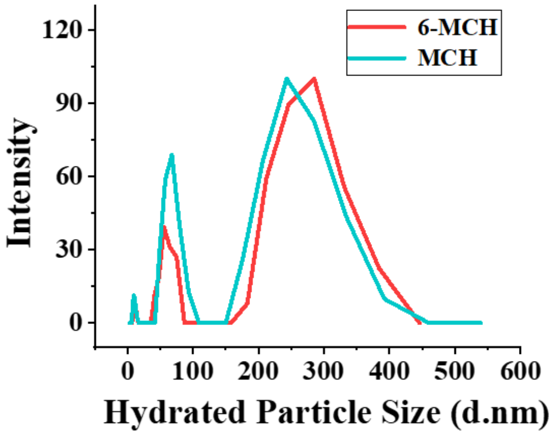

Figure S5. Hydrated particle size of 6-MCH-functionalized Au NPs and MCH-functionalized Au NPs.

S7. Comparison of current intensity of Au NPs assemblies with MCH ($C_{MCH}/C_{Au\ NPs} \approx 1.2 \times 10^3$) at different time.

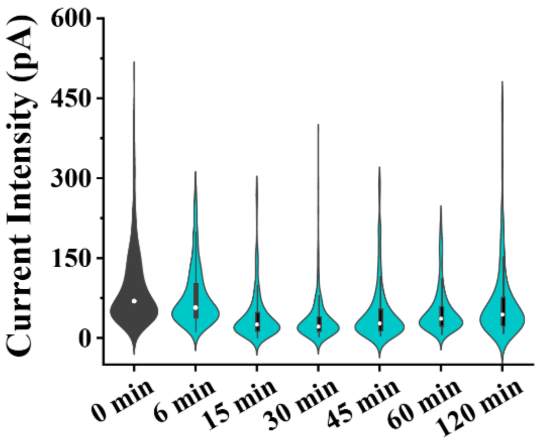

Figure S6. Violin plots of current intensity of Au NPs assemblies with MCH ($C_{MCH}/C_{Au\ NPs} \approx 1.2 \times 10^3$) at different time.

Reference

[1] Bai, Y. Y.; Yang, Y. J.; Xu, Y.; Yang, X. Y.; Zhang, Z. L., Current Lifetime of Single-Nanoparticle Electrochemical Collision for in Situ Monitoring Nanoparticles Agglomeration and Aggregation. *Anal. Chem.* **2023**, *95*, 4429-4434.

[2] Frens, G., Controlled Nucleation for the Regulation of the Particle Size in Monodisperse Gold Suspensions. *Nat. Phys. Sci.* **1973**, *241*, 20-22.

[3] Bai, Y. Y.; Feng, Z. T.; Yang, Y. J.; Yang, X. Y.; Zhang, Z. L., Current Lifetime of Single-Nanoparticle Collision for Sizing Nanoparticles. *Anal. Chem.* **2022**, *94*, 1302-1307.

[4] Li, Y. T.; Zhang, S. H.; Wang, L.; Xiao, R. R.; Liu, W.; Zhang, X. W.; Zhou, Z.; Amatore, C.; Huang, W. H., Nanoelectrode for Amperometric Monitoring of Individual Vesicular Exocytosis inside Single Synapses. *Angew. Chem. Int. Ed.* **2014**, *53*, 12456-12460.
